# Meeting Report from the Genomic Standards Consortium (GSC) Workshop 8

**DOI:** 10.4056/sigs.1022942

**Published:** 2010-08-20

**Authors:** Nikos Kyrpides, Dawn Field, Peter Sterk, Renzo Kottmann, Frank Oliver Glöckner, Lynette Hirschman, George M. Garrity, Guy Cochrane, John Wooley

**Affiliations:** 1 DOE Joint Genome Institute, Walnut Creek, CA, USA; 2 NERC Center for Ecology and Hydrology, Oxford, UK; 3 Wellcome Trust Sanger Institute, Wellcome Trust Genome Campus, Hinxton, UK; 4 Microbial Genomics Group, Max Planck Institute for Marine Microbiology and Jacobs University Bremen, Germany; 5 Information Technology Center, The MITRE Corporation, USA; 6 Department of Microbiology and Molecular Genetics, Michigan State University, East Lansing, MI USA; 7 European Molecular Biology Laboratory (EMBL) Outstation, European Bioinformatics Institute (EBI), Wellcome Trust Genome Campus, Hinxton, UK; 8 University of California San Diego, La Jolla, USA

## Abstract

This report summarizes the proceedings of the 8th meeting of the Genomic Standards Consortium held at the Department of Energy Joint Genome Institute in Walnut Creek, CA, USA on September 9-11, 2009. This three-day workshop marked the maturing of Genomic Standards Consortium from an informal gathering of researchers interested in developing standards in the field of genomic and metagenomics to an established community with a defined governance mechanism, its own open access journal, and a family of established standards for describing genomes, metagenomes and marker studies (i.e. ribosomal RNA gene surveys). There will be increased efforts within the GSC to reach out to the wider scientific community via a range of new projects. Further information about the GSC and its activities can be found at http://gensc.org/.

## Introduction

The Genomic Standards Consortium (GSC) is an international working body with the mission of working towards richer descriptions of our collection of genomes and metagenomes through the development of standards and tools for supporting compliance and exchange of contextual information [1]. The GSC has over 100 members spread across the major bioinformatics and genome sequencing centers in the world. We are now in an era of “mega-sequencing” projects that include funded projects like the Genomic Encyclopedia of Bacteria and Archaea (GEBA) project [2] and the Human Microbiome Project [3], with many more visionary projects on the horizon. Data generated by these projects hold the promise of unparalleled insights into fundamental questions across a range of fields including evolution, ecology, environment biology, health and medicine. Because the pace of genomic and metagenomic sequencing projects is increasing so rapidly [4], the role of standards has taken on a central role in scientific progress and data sharing, even more so given the widespread application of ultra-high-throughput methods. In this context, the GSC has been organizing workshops on a regular basis during which participants have the opportunity to advance its core projects, propose new ones and establish linkages between the GSC and relevant scientific projects.

This report summarizes the proceedings of the 8^th^ workshop of the GSC held September 9-11, 2009 at the DOE Joint Genome Institute in Walnut Creek, CA, United States.

The workshop was recorded on video by JGI and all talks are accessible via SciVee at URL http://www.scivee.tv/node/12786. A meeting report that provides further details on the meeting has appeared in the JGI newsletter The Primer [5]. Aspects of the meeting were also been covered in the press [6].

## The GSC 8 Workshop

The goal of the workshop was to review progress on all GSC projects and activities, to launch the Minimal Information about an ENvironmental Sequence (MIENS) specification, and to revisit in more detail the growing need for new standards for generating and comparing genomic and metagenomic annotations. The main meeting was preceded by an ISA-Tab/GCDML alignment Workshop attending by about 20 participants. This meeting led to an expanded and refined set of requirements for the vision of the GSC Genome Catalogue [4]. The proposal was put forward that such a catalogue could be built upon a collaborative infrastructure that combined the GOLD database [7,8] the ISA Infrastructure [8] to support integration of multi-omic metadata and the INSDC as the final archive of all data. A full description of these requirements was added to the GSC wiki for further discussion.

The first session of the main meeting included updates on all of the GSC core projects and set the stage for the rest of the workshop. Topics of the presentations included the GSC eJournal Standards in Genomic Sciences, the launch of the National Science Foundation Research Coordination Network RCN4GSC (2009-2013), GSC Governance, the GSC family of metadata standards: MIGS/MIMS/MIENS (including curation efforts, a vision for a Genomes and Metagenomes (GEM) Catalogue and the latest MIENS specification and submission of metadata to INSDC) and ideas for a GSC Global Genome Census. A highlight was the decision of the Finishing standards group represented by Patrick Chain (DOE JGI) to work under the umbrella of the GSC as the authoritative community with the goal of promoting standards in this domain [9].

The second session of the day was dedicated to flash talks from adopters of GSC standards and future mega sequencing projects. These included talks from representatives of ‘The Genomic Encyclopedia of Bacteria and Archaea’ (GEBA), ‘The Human Microbiome Project’ (HMP), ‘The Terragenome Initiative’ and ‘The Tara-Oceans Project’. During his talk, the leader of the HMP, George Weinstock, urged the GSC to reach out and promote the adoption of the GSC standards by other communities. One way to help achieve this was agreed during discussions and involves working closely with major projects such as the HMP and The Terragenome Initiative.

In the next session, a roadmap was presented for the Metagenomics, Metadata and MetaAnalysis, Models and MetaInfrastructure (M5) initiative. This working group was established during the GSC Special Interest Group meeting at the ISMB meeting in Stockholm in June 2009 [10] to address the computational needs of the large scale sequencing projects. Further discussions during the course of the meeting led to agreements to work on metagenomic data exchange between MG-RAST [11] and IMG [12], the development of a common workflow language, and a data-file exchange standard.

The finalization of the MIENS checklist was another major goal of the workshop. This was achieved during the course of the meeting in breakout sessions and version 2.1 of the MIGS/MIMS/MIENS checklist was presented at the end of day 2.

Day two included three sessions and started with the consideration of genomic annotations “Unifying concepts in genomic annotation: from SOPs to standards”. This launched a working group to take forward a minimum information checklist for genome annotations and for gene calling to be led by Nikos Kyrpides (DOE JGI). In addition, the idea of consensus annotation was proposed and discussed and has since been taken forward by Owen White (University of Maryland) as the Critical Assessment of Functional Annotation Experiment (CAFAE). This was followed by breakout sessions on MIENS and M5 and a formal session dedicated to the vision of the SIGS journal.

Day three started with a session that included outreach to other communities and including introductions to the International Society for Biocuration by Pascale Gaudet (Northwestern) and the BioSharing initiative by Susanna Sansone (EBI). The final session of the meeting was dedicated to progressing the GSC roadmap, wrapping up the discussions and listing actions.

## Meeting Outcomes

The main outcomes of the meeting are listed below and represent further steps in the maturation of the GSC from an informal gathering of scientists to a more formal ‘voice’ for the genomics and metagenomics community.

**Launched:**
- RCN4GSC launched with first face-to-face meeting of Steering Committee
- Metagenomics, Metadata, MetaAnalysis, Models and MetaInfrastructure (M5) initiative
- Microbial Earth initiative
- GSC involvement in the BioSharing [13] (http://biosharing.org) Policy Forum
- Development of a new vision for the GSC Genome Catalogue

**Agreements:**
- MIGS/MIMS/MIENS version 2.1 and incorporation of these standards into the INSDC databases
- Environmental Markup Language (EML) will integrate GCDML

**Future meetings:**
- January 4, 2010: Workshop at the Pacific Symposium on Biocomputing in Hawaii
- March 28-30, 2010: 9^th^ Genomic Standards Consortium Workshop at the J. Craig Venter Institute with open registration
- Submission of a proposal for an ISMB M3/BioSharing Special Interest Group meeting in Boston 2010 July 9-10.

**Governance:**
- GSC Board, SIGS Editorial Board, RCN Steering Committee formed and now active

**New working groups:**
- Minimum information about a genome annotation and Gene Calling

## Conclusions

This was the second GSC meeting to be held in the US and the first to be held at a major sequencing center. It was the first to be recorded as video and the first to be covered by the press. The success of this meeting set the stage for the subsequent GSC 9 which was held at the JCVI in April 2009. Outcomes included further engagement with a range of related communities and significant progress on GSC core projects.

## References

[1] Field D Garrity GM Sansone SA Sterk P Gray T Kyrpides N Hirschman L Glockner FO Kottmann R Angiuoli S Meeting report: the fifth Genomic Standards Consortium (GSC) workshop. OMICS 2008; 12:109-113 10.1089/omi.2008.A3B3 18564915

[2] Wu D Hugenholtz P Mavromatis K Pukall R Dalin E Ivanova NN Kunin V Goodwin L Wu M Tindall BJ A phylogeny-driven genomic encyclopaedia of Bacteria and Archaea. Nature 2009; 462:1056-1060 10.1038/nature08656 20033048 PMC3073058

[3] Nelson KE Weinstock GM Highlander SK Worley KC Creasy HH Wortman JR Rusch DB Mitreva M Sodergren E Chinwalla AT A catalog of reference genomes from the human microbiome. Science 2010; 328:994-999 10.1126/science.1183605 20489017 PMC2940224

[4] Field D Garrity G Gray T Morrison N Selengut J Sterk P Tatusova T Thomson N Allen MJ Angiuoli SV The minimum information about a genome sequence (MIGS) specification. Nat Biotechnol 2008; 26:541-547 10.1038/nbt1360 18464787 PMC2409278

[5] Santos Ballon M. Setting the Standards. The Primer 2009.

[6] Marx V. Genome Standards Consortium Evolves and Expands to Keep Pace with Rapid Advances in the Field. Bioinform. 2009.

[7] Liolios K Mavromatis K Tavernarakis N Kyrpides NC The Genomes On Line Database (GOLD) in 2007: status of genomic and metagenomic projects and their associated metadata. Nucleic Acids Res 2008; 36(Database issue):D475-D479 10.1093/nar/gkm884 17981842 PMC2238992

[8] Rocca-Serra P Brandizi M Maguire E Sklyar N Taylor C Begley K Field D Harris S Hide W Hofmann O ISA software suite: supporting standards-compliant experimental annotation and enabling curation at the community level. Bioinformatics 2010 10.1093/bioinformatics/btq415 PMC2935443 20679334

[9] Chain PS Grafham DV Fulton RS Fitzgerald MG Hostetler J Muzny D Ali J Birren B Bruce DC Buhay C Genomics. Genome project standards in a new era of sequencing. Science 2009; 326:236-237 10.1126/science.1180614 19815760 PMC3854948

[10] Field D Friedberg I Sterk P Kottmann R Glöckner FO Hirschman L Garrity GM Cochrane G Wooley J Gilbert J Meeting Report: “Metagenomics, Metadata and Meta-analysis” (M3) Special Interest Group at ISMB 2009. Stand Genomic Sci 2009; 1:278-282 10.4056/sigs.641096 PMC3035241 21304668

[11] Meyer F Paarmann D D'Souza M Olson R Glass EM Kubal M Paczian T Rodriguez A Stevens R Wilke A The metagenomics RAST server - a public resource for the automatic phylogenetic and functional analysis of metagenomes. BMC Bioinformatics 2008; 9:386 10.1186/1471-2105-9-386 18803844 PMC2563014

[12] Markowitz VM Chen IM Palaniappan K Chu K Szeto E Grechkin Y Ratner A Anderson I Lykidis A Mavromatis K The integrated microbial genomes system: an expanding comparative analysis resource. Nucleic Acids Res 2010; 38(Database issue):D382-D390 10.1093/nar/gkp887 19864254 PMC2808961

[13] Field D Sansone SA Collis A Booth T Dukes P Gregurick SK Kennedy KL Kolar P Kolker E Maxon M 'Omics Data Sharing. Science 2009; 326:234-236 10.1126/science.1180598 19815759 PMC2770171

